# The relationship between visceral adipose tissue and osteoarthritis among older adults: evidence from the NHANES 1999–2018

**DOI:** 10.3389/fnut.2025.1526377

**Published:** 2025-02-05

**Authors:** Wei Huang, Yin-sheng Xiao, Yu-hang Zou, Lu-qun Zhong, Gui-qiong Huang

**Affiliations:** Huizhou Hospital of Guangzhou University of Chinese Medicine, Huizhou, China

**Keywords:** osteoarthritis, obesity, visceral adiposity index, cross-sectional study, NHANES

## Abstract

**Objectives:**

This study aimed to assess any possible links between visceral fat accumulation and an elevated prevalence of osteoarthritis (OA) in the elderly.

**Methods:**

3,779 subjects aged 65 years or older from the National Health and Nutrition Examination Survey (NHANES) 1999–2018 were finally included, of whom 516 had OA. The identification of patients diagnosed with OA was conducted using questionnaire data. The visceral adiposity index (VAI) was computed utilizing body mass index (BMI), waist circumference (WC), triglycerides (TG), and high-density lipoprotein cholesterol (HDL-C). To investigate the relationship between VAI and OA, weighted multivariable logistic regression analysis, restricted cubic spline (RCS), subgroup analyses, and interaction tests were carried out.

**Results:**

Multivariate logistic regression analysis showed that the increase in VAI is accompanied by an increased prevalence of OA after fully correcting for variables. The top quartile of VAI had a prevalence that was 110% higher than the lowest quartile. A non-linear positive correlation between VAI and OA was found in the RCS.

**Conclusion:**

This study suggests a potential correlation between elevated VAI and increased prevalence of OA in older adults, and that lowering VAI may have an impact on the prevalence of OA.

## 1 Introduction

OA is a common degenerative disorder of the joints and the primary musculoskeletal source of mobility problems in the elderly (1, 2). It is characterized by degeneration of the articular cartilage and changes in the joint structure, leading to pain, stiffness and functional limitations (3). One-third of individuals aged 65 and older are afflicted with the disease, which is particularly prevalent among this age group worldwide (4, 5). Most people present with imaging evidence of OA at the age of 65 years, and about 80% of people with OA over the age of 75 have imaging evidence of OA (6). The health and economic burden of OA is increasing in light of an aging global population (7). Statistically, OA results in approximately $47.9 billion in personal healthcare expenditures in the United States (8). Therefore, exploration of OA prevalence factors in the elderly population and early intervention is essential.

In recent years, the potential relationship between visceral adipose tissue (VAT) and OA has attracted the attention of many researchers as obesity rates continue to rise. VAT not only plays a key role in energy storage, but also significantly influences the metabolic and inflammatory status of the whole body through the secretion of a variety of bioactive molecules such as adipokines and cytokines (9–11). Studies have shown a correlation between increased VAT and an increased prevalence of developing OA, which may be related to the chronic inflammatory state and metabolic disturbances caused by VAT (12–14). VAT secretes pro-inflammatory cytokines, such as tumor necrosis factor-alpha (TNF-alpha) and interleukin 6 (IL-6), which have been shown to be closely associated with the pathological process of OA. These inflammatory factors not only promote chondrocyte apoptosis and cartilage matrix degradation, but also exacerbate localized inflammatory responses in the joints, thereby accelerating the process of joint degeneration (15, 16). In addition, VAT has been associated with metabolic disorders such as insulin resistance and metabolic syndrome, which have also been found to be linked to the development of OA (17). For example, Yoshinori Asou et al. found that a high-fat diet not only promoted osteoid formation through mechanical load-dependent and independent mechanisms, but also increased chondrocyte apoptosis and was associated with inflammatory and metabolic changes in the adipose pad, which in turn affected the progression of OA (16). Kelsey H. Collins et al. found that lipids in adipose tissue-deficient protective against OA in dystrophic mice even on a high-fat diet, that adipose tissue affects joint degeneration through paracrine signaling rather than body weight, and that adipokines may be associated with levels of proinflammatory mediators associated with hepatic steatosis (18).

Previous researchers have mainly investigated the relationship between body weight, BMI, metabolic score of visceral fat (METS-VF), body roundness index (BRI), and OA, and have found that there is a certain positive correlation between these indices and OA (19–22). This study focuses on the relationship between a new gender-specific index, the VAI, and the prevalence of OA in older adults. Studies have shown that there is an association between BMI and the extent of visceral and subcutaneous adipose tissue (23). Assisting in the evaluation of VAT accumulation and function impairment, The VAI consists of BMI, WC, TG, and HDL-C (24). Previous studies have established a correlation between elevated levels of VAI and the development of kidney stones, erectile dysfunction, and cardiometabolism (25–27). In the elderly, however, the relationship between VAI and the prevalence of OA is not recognized.

The purpose of our study was to provide insight into the possible association between VAI and OA in the elderly population. We hypothesized that elevated VAI might have an effect on the prevalence of OA.

## 2 Materials and methods

### 2.1 Study population

The information we utilized was obtained from the NHANES database, an annual monitoring system that examines 5,000 individuals nationwide. The database encompasses various types of data, including questionnaires and limited access, demographic, dietary, examination, laboratory, and questionnaire. The research study received approval from the National Ethical Review Board for Health Statistics Research, and all participants provided informed consent by completing informed consent forms.

We derived data from ten consecutive cycles of NHANES from 1999–2018. The weight variables for our study were chosen as WTSAF4YR and WTSAF2YR. The weights for 1999–2000 and 2001–2002 were computed using the formula 2/10 × WTSAF4YR, and for 2003–2018 the weights were computed using the formula 1/10× WTSAF2YR.

**Figure 1:**
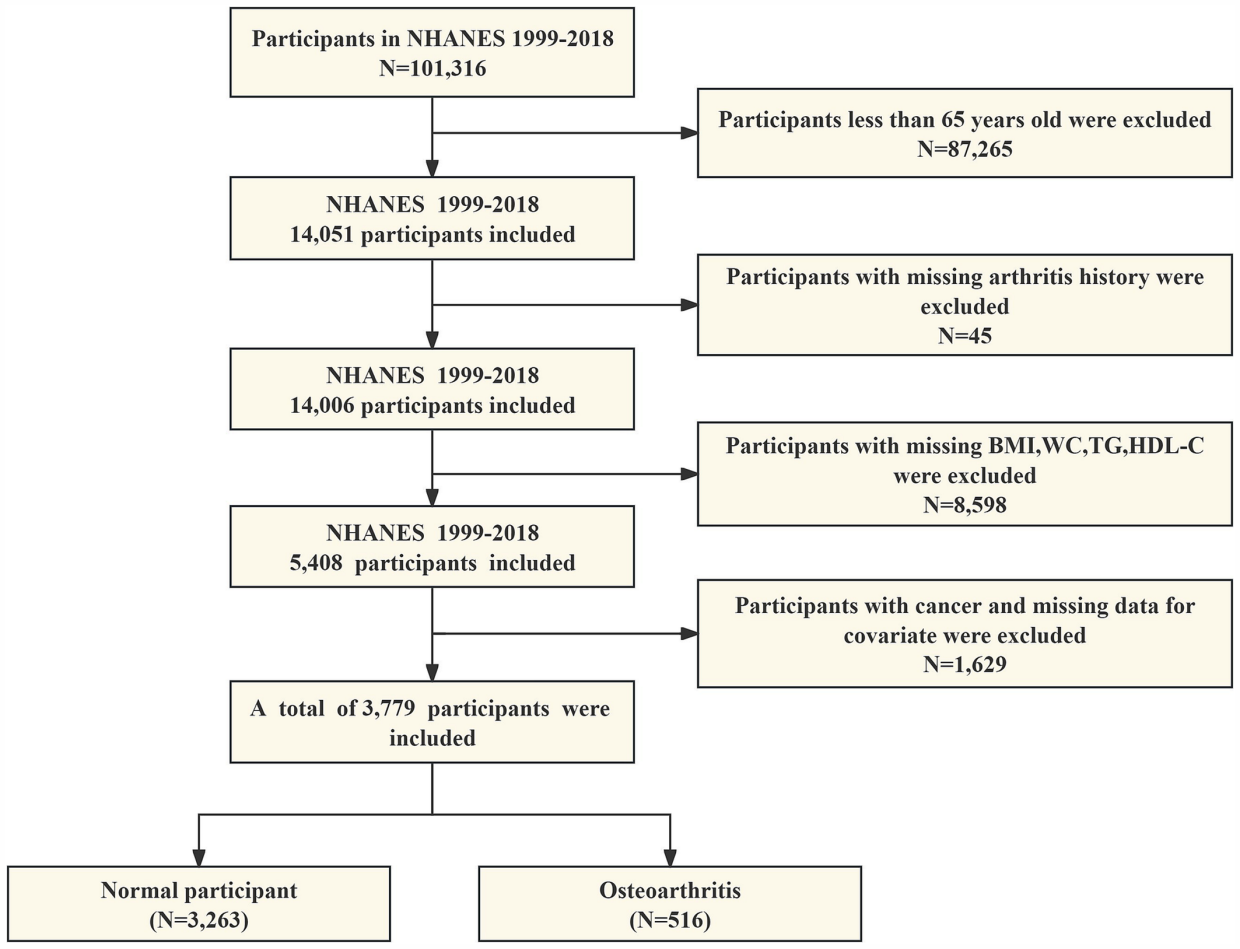
A flow chart of participants screening in NHANES 1999–2018.

Our study ruled out the following: (1) adults younger than 65 years of age; (2) participants with absent data on arthritis; (3) participants with absent data on BMI, WC, TG, and HDL-C; and (4) participants with cancer and missing data on all covariates. As shown in Figure 1, 3,779 eligible individuals aged ≥65 years were ultimately enrolled in this study.

### 2.2 Measurement of VAI

An assessment of visceral adiposity function, the VAI index is a sex-specific metric composed of WC, BMI, TG, and HDL-C. Higher VAI levels predicted severe visceral obesity. The VAI was computed utilizing the subsequent formula:

$$\begin{array}{l} \mathrm{Males}=\frac{WC\left(\mathrm{cm}\right)}{39.68+1.88\times BMI\left(\mathrm{kg}/m^{2}\right)}\times \frac{TG\left(\mathrm{mmol}/L\right)}{1.03}\times \\ \frac{1.31}{HDL\left(\mathrm{mmol}/L\right)} \end{array}$$

$$\begin{array}{l} \mathrm{Females}=\frac{WC\left(\mathrm{cm}\right)}{36.58+1.89\times BMI\left(\mathrm{kg}/m^{2}\right)}\times \frac{TG\left(\mathrm{mmol}/L\right)}{0.81}\times \\ \frac{1.51}{HDL\left(\mathrm{mmol}/L\right)} \end{array}$$

The VAI was treated as a continuous variable in this analysis, and for subsequent analyses, participants were divided into four groups according to their VAI quartiles.

### 2.3 Definition of OA

Arthritis diagnosis information was extracted from self-reported questionnaire data. Participants were queried regarding any previous arthritis diagnosis they may have received from a physician or other healthcare practitioner. If the answer was affirmative, they were asked to further describe the type of arthritis (OA, rheumatoid arthritis, psoriatic arthritis, or other type of arthritis). Studies have shown that there is a concordance between self-reported arthritis and clinically diagnosed arthritis (28).

### 2.4 Covariates

Covariates in our study embraced age (years), sex (male, female), race (Mexican American, Non-Hispanic White person, Non-Hispanic Black person, others), marital status (married or partnered, never married, widowed or divorced or separated), education attainment (below high school, high school, above high school), smoking status (never, former, current), alcohol consumption (whether greater than 12 drinks in 1 year), hypertension (yes, no), diabetes mellitus (yes, no), total cholesterol (TC), Low-density lipoprotein cholesterol (LDL-C), blood calcium (Ca), blood phosphorus (P), blood urea nitrogen (BUN), uric acid (UA), and blood creatinine (Cr).

### 2.5 Statistical methods

All the analyses were conducted using R (version 4.2.3). Weighted means and standard deviations are utilized to calculate continuous variables, whereas weighted percentages are employed to calculate categorical variables. We used chi-square tests and t-tests, respectively, to compare categorical and continuous variables within the VAI index quartile classifications. The correlation between OA and VAI was examined through the implementation of weighted multivariate logistic regression models. In Model 1, covariates were unadjusted. In Model 2, adjustments were made for age, sex, and race. In model 3, adjustments were made for age, sex, race, education attainment, marital status, smoking, alcohol consumption, hypertension, diabetes, cholesterol, LDL-C, Ca, P, BUN, UA, and Cr. In addition, we used VAI as quartiles for grouping. RCS was utilized to probe the nonlinear relationships. In the end, we conducted stratified analyses and interaction tests by age (<75/≥75 years), sex (male/female), BMI (normal/overweight/obesity), race (Mexican American, Non-Hispanic White person, Non-Hispanic Black person, others), education attainment (below high school, high school, above high school), hypertension, and diabetes. The *p*-value of the statistical test was less than 0.05, indicating statistical significance.

## 3 Results

### 3.1 General characteristics of the participants

A total of 3,779 participants were enrolled in this study, with a mean age of 72.39 ± 5.58, including 55.68% females, 78.68% Non-Hispanic White person, and a prevalence of OA of 14.75%. Table 1 shows the baseline characteristics of the participants according to VAI quartiles. VAI index values for quartiles 1 to 4 were ≤ 1.01, 1.01–1.62, 1.62–2.57, and ≥ 2.57, respectively. Differences among the VAI quartiles were statistically significant for sex, race, education attainment, alcohol consumption, hypertension, diabetes mellitus, weight, BMI, WC, total cholesterol, LDL-C, HDL-C, TG, Ca, and UA (*p* < 0.05).

**Table 1:** Weighted demographic characteristics of all participants.

Characteristic	Overall, *N* = 3,779 (100%)	Q1, *N* = 945 (25%)	Q2, *N* = 945 (25%)	Q3, *N* = 944 (24%)	Q4, *N* = 945 (26%)	*P* value
VAI	2.01 ± 1.47	0.70 ± 0.21	1.30 ± 0.18	2.03 ± 0.27	3.96 ± 1.48	<0.001
Age (years)	72.39 ± 5.58	72.60 ± 5.60	72.32 ± 5.67	72.69 ± 5.56	71.98 ± 5.50	0.10
Age, year (%)						0.2
below 75	2,284 (65.29%)	549 (63.12%)	561 (65.84%)	571 (63.49%)	603 (68.54%)	
above 75	1,495 (34.71%)	396 (36.88%)	384 (34.16%)	373 (36.51%)	342 (31.46%)	
Sex (%)						<0.001
Male	1,884 (55.68%)	567 (53.11%)	482 (46.14%)	420 (38.28%)	415 (39.59%)	
Female	1,895 (55.68%)	378 (46.89%)	463 (53.86%)	524 (61.72%)	530 (60.41%)	
Race (%)						<0.001
Mexican American	577 (4.20%)	89 (2.94%)	139 (4.41%)	166 (4.92%)	183 (4.55%)	
Non-Hispanic White	2,034 (78.68%)	461 (75.32%)	521 (80.01%)	492 (76.11%)	560 (83.01%)	
Non-Hispanic Black	636 (8.44%)	271 (14.24%)	162 (8.16%)	125 (7.22%)	78 (4.20%)	
Others	532 (8.68%)	124 (7.50%)	123 (7.42%)	161 (11.75%)	124 (8.23%)	
Education level (%)						0.001
Below high school	1,378 (23.62%)	294 (20.63%)	340 (23.52%)	362 (24.94%)	382 (25.40%)	
High school	913 (27.28%)	204 (22.64%)	219 (26.44%)	225 (27.92%)	265 (32.03%)	
Above high school	1,488 (49.10%)	447 (56.73%)	386 (50.04%)	357 (47.14%)	298 (42.57%)	
Marital status (%)						0.6
Married/Living with partner	2,176 (63.09%)	550 (64.41%)	551 (63.44%)	544 (63.97%)	531 (60.64%)	
Never married	127 (2.63%)	43 (3.42%)	29 (2.49%)	28 (2.23%)	27 (2.36%)	
Widowed/Divorced/Separated	1,476 (34.28%)	352 (32.16%)	365 (34.06%)	372 (33.80%)	387 (37.00%)	
Smoking status (%)						0.2
Never smoker	1,832 (48.32%)	448 (47.84%)	476 (48.42%)	474 (51.72%)	434 (45.58%)	
Former smoker	1,553 (42.85%)	385 (43.20%)	374 (44.75%)	383 (39.46%)	411 (43.77%)	
Current smoker	394 (8.83%)	112 (8.96%)	95 (6.83%)	87 (8.82%)	100 (10.65%)	
Alcohol consumption (%)						0.031
<12 drinks	1,651 (43.44%)	385 (39.83%)	397 (41.79%)	432 (43.60%)	437 (48.39%)	
12 drinks+	2,128 (56.56%)	560 (60.17%)	548 (58.21%)	512 (56.40%)	508 (51.61%)	
Hypertension (%)						<0.001
No	1,060 (31.01%)	307 (39.82%)	282 (32.79%)	247 (27.42%)	224 (24.03%)	
Yes	2,719 (68.99%)	638 (60.18%)	663 (67.21%)	697 (72.58%)	721 (75.97%)	
Diabetes mellitus (%)						<0.001
No	2,650 (74.34%)	735 (84.20%)	694 (76.67%)	635 (71.14%)	586 (65.44%)	
Yes	1,129 (25.66%)	210 (15.80%)	251 (23.33%)	309 (28.86%)	359 (34.56%)	
Weight (kg)	78.36 ± 17.91	73.52 ± 16.94	77.39 ± 17.59	80.34 ± 18.32	82.19 ± 17.62	<0.001
Height (cm)	165.26 ± 9.86	166.01 ± 9.61	164.85 ± 10.35	164.89 ± 9.50	165.27 ± 9.91	0.2
WC (cm)	101.47 ± 13.91	95.81 ± 13.63	100.51 ± 12.92	103.60 ± 13.78	105.92 ± 13.22	<0.001
BMI (kg/m2)	28.59 ± 5.64	26.57 ± 5.25	28.37 ± 5.53	29.43 ± 5.82	29.98 ± 5.37	<0.001
TC (mmol/L)	5.04 ± 1.13	4.93 ± 1.02	5.00 ± 1.08	5.02 ± 1.15	5.21 ± 1.22	0.003
HDL-C (mmol/L)	1.48 ± 0.46	1.88 ± 0.51	1.53 ± 0.35	1.36 ± 0.29	1.14 ± 0.27	<0.001
TG (mmol/L)	1.46 ± 0.72	0.76 ± 0.21	1.14 ± 0.22	1.52 ± 0.29	2.38 ± 0.63	<0.001
LDL-C (mmol/L)	2.90 ± 0.97	2.70 ± 0.84	2.94 ± 0.93	2.97 ± 1.00	2.99 ± 1.08	<0.001
P (mmol/L)	1.17 ± 0.17	1.17 ± 0.16	1.17 ± 0.17	1.17 ± 0.17	1.18 ± 0.17	0.9
Total calcium (mmol/L)	2.35 ± 0.09	2.34 ± 0.08	2.36 ± 0.10	2.36 ± 0.10	2.36 ± 0.09	<0.001
BUN (mmol/L)	6.05 ± 2.41	6.03 ± 2.12	6.04 ± 2.36	5.96 ± 2.39	6.18 ± 2.74	0.4
UA (umol/L)	341.44 ± 86.22	323.52 ± 79.71	332.33 ± 83.11	341.47 ± 86.18	367.68 ± 89.12	<0.001
Cr (umol/L)	86.04 ± 35.83	85.23 ± 31.80	86.16 ± 44.40	84.74 ± 30.00	87.91 ± 35.09	0.2
OA						0.002
Non-OA	3,263 (85.25%)	839 (90.22%)	807 (84.28%)	817 (84.71%)	800 (81.88%)	
OA	516 (14.75%)	106 (9.78%)	138 (15.72%)	127 (15.29%)	145 (18.12%)	

VAI, Visceral adiposity index; WC, waist circumference; BMI, Body Mass Index; TC, Total Cholesterol; HDL, High-Density Lipoprotein; TG, Triglycerides; LDL, Low-Density Lipoprotein; P, Blood phosphorus; BUN, Blood urea nitrogen; UA, Uric acid; Cr, Blood creatinine; Mean ± standard deviation (SD) for continuous; *n* (%) for categorical.

### 3.2 Relationship between VAI and OA

Table 2 shows the results of multivariate logistic regression. In model 1, which was not adjusted for any covariates, with each unit increase in VAI, there was a corresponding 11% increase in the prevalence of OA (odds ratio (OR) = 1.11, 95% confidence intervals (CI): 1.04–1.19, *p* = 0.002). In model 2 adjusted for age, sex and race, the findings remained consistent (OR = 1.10, 95% CI: 1.03–1.18, *p* = 0.006). After further adjusting for the covariates of education level, marital status, smoking status, drinking habits, hypertension, diabetes mellitus, BUN, Ca, P, LDL-C, TC, Cr, and UA, the positive association between VAI and the prevalence of OA remained significant in Model 3 (OR = 1.11, 95% CI: 1.03–1.19, *p* = 0.004). In addition, we converted VAI, which was originally a constant variable, to quartiles for sensitivity analysis. In Model 1, the prevalence of OA increased by 72, 66, and 104% in the second, third, and fourth quartiles (Q2, Q3, and Q4), respectively, compared with the lowest quartile (Q1). In Models 2 and 3, these prevalences increased by 69, 58, and 97% and 78, 71, and 110%, respectively. This suggests that the prevalence of OA increases significantly as the value of VAI increases.

**Table 2:** Association between visceral adiposity index and osteoarthritis.

	Model 1			Model 2			Model 3		
Characteristic	OR	95% CI	*p*-value	OR	95% CI	*p*-value	OR	95% CI	*p*-value
VAI	1.11	1.04, 1.19	0.002	1.10	1.03, 1.18	0.006	1.11	1.03, 1.19	0.004
VAI (quartile)									
Q1		Ref			Ref			Ref	
Q2	1.72	1.18, 2.50	0.005	1.69	1.16, 2.46	0.007	1.78	1.22, 2.61	0.003
Q3	1.66	1.13, 2.43	0.010	1.58	1.06, 2.36	0.024	1.71	1.13, 2.59	0.011
Q4	2.04	1.40, 2.97	<0.001	1.97	1.34, 2.88	<0.001	2.10	1.44, 3.07	<0.001
*P* for trend		<0.001			0.002			<0.001	

OR = Odds Ratio, CI = Confidence Interval. Model 1, No covariates were adjusted, Model 2, Adjusted for sex, age, and race, Model 3, Adjusted for sex, age, race, education attainment, marital status, smoking status, alcohol consumption, hypertension, diabetes, BUN, Ca, P, LDL-C, TC, Cr, and UA.

Given that Table 2 presents only the results of multivariate logistic regression analyses, further incorporation of the results of other statistical methods (e.g., RCS analyses, subgroup analyses, and tests of interactions) is necessary for a more comprehensive assessment of the complexity of the association between VAI and OA prevalence. Although Model 3 has been adjusted for multiple covariates, there may still be unconsidered confounders present that may have an impact on the association between VAI and OA prevalence. In addition, due to the cross-sectional design of the study, causality could not be established and only the association between VAI and OA prevalence could be illustrated.

### 3.3 Nonlinear relationship between OA and VAI

**Figure 2:**
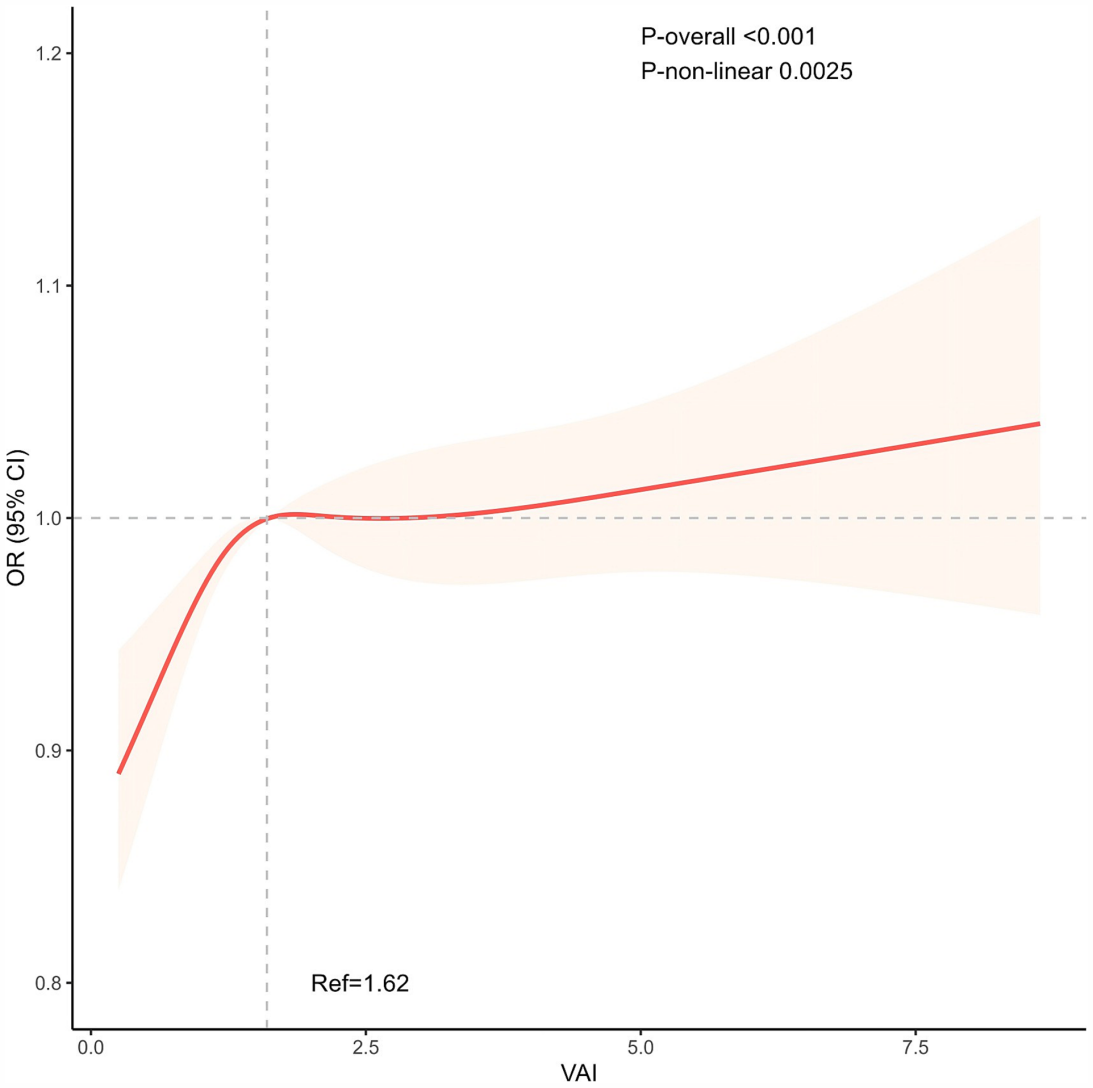
The non-linear positive association between VAI and OA. The median (VAI = 1.62) was used as the reference point. VAI, Visceral adiposity index.

As shown in Figure 2, we visualized the relationship between OA and VAI using a RCS curve with the median (VAI = 1.62) as the reference point. A positive nonlinear correlation was discovered between OA and VAI prevalence. The overall correlation was statistically significant (*P* overall <0.001). The curves showed that the prevalence of OA increased rapidly at lower values of VAI and slowed down at higher values (*P* nonlinear = 0.0025).

### 3.4 Subgroup analyses and interaction tests

**Figure 3:**
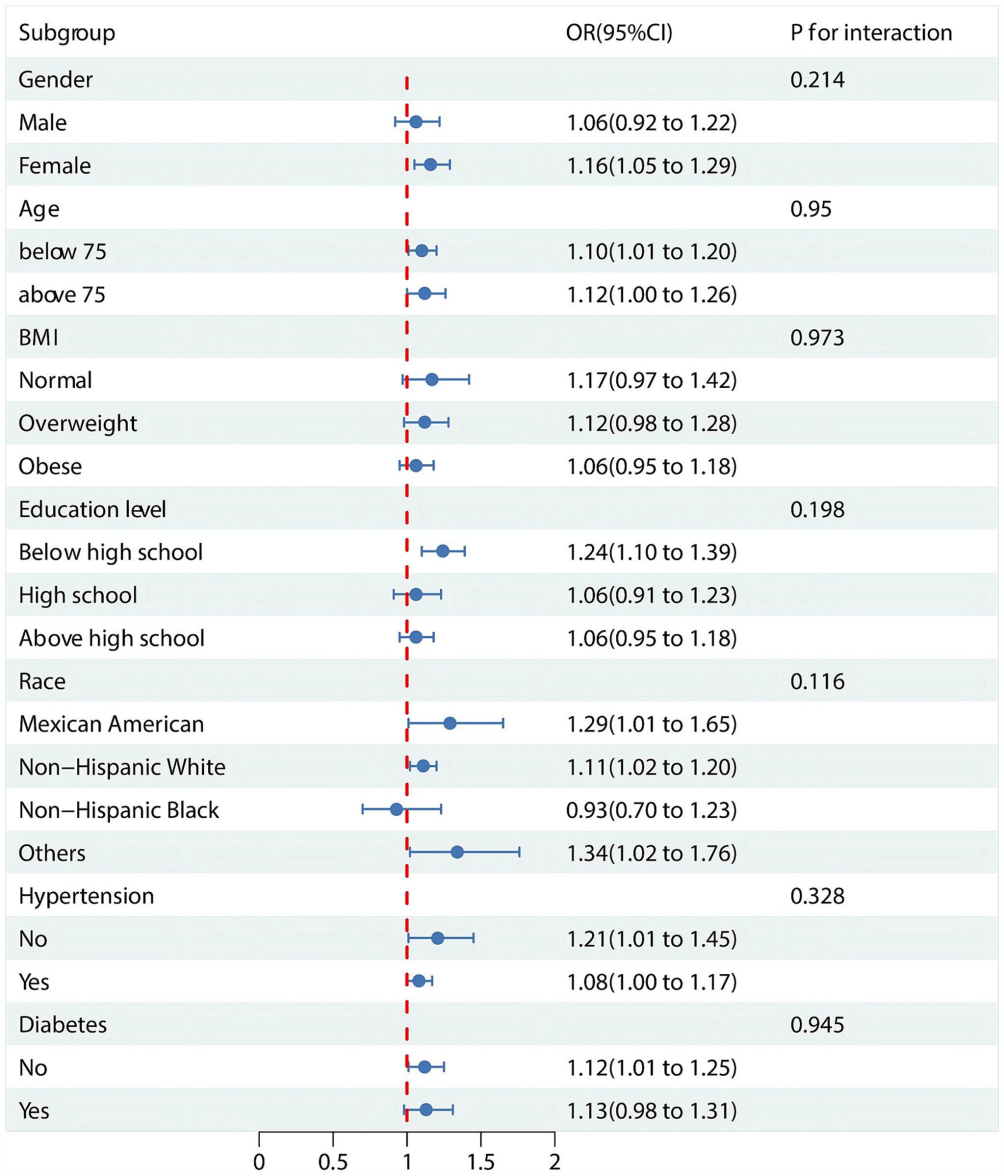
Subgroup analysis and interaction tests for the association between VAI and OA. OR: odds ratio.

The subgroup analyses and interaction tests are detailed in Figure 3. In subgroup analyses stratified by sex, age, race, education attainment, BMI, diabetes mellitus, and hypertension, we found that the prevalence of OA did not correlate with elevated VAI levels in some subgroups. Overall, There was no statistical significance to this correlation (*p* > 0.05) for males, BMI, high school education and above, Non-Hispanic Black person, and diabetes. In addition, interaction tests showed no significant effect of sex, age, race, education attainment, BMI, diabetes, and hypertension on this association (*p* > 0.05 for all interactions).

## 4 Discussion

The article is an original research article that used data from the NHANES and utilized statistical methods such as weighted multivariate logistic regression analysis, RCS, subgroup analysis, and interaction tests. The study described in detail the selection and exclusion criteria of the subjects and analyzed them based on the actual data collected. The findings include the results of multivariate logistic regression analysis and the relationship between VAI and OA. The article also discusses the results of the study, explores possible associations between VAI and OA, and points out the limitations of the study and directions for future research.

Our study is the first to utilize the NHANES database to report higher levels of VAI in elderly OA patients than in healthy individuals. Several previous studies have investigated the connection between VAI and OA in diverse aspects. Visser, A. Willemien Visser, et al. discovered a correlation between VAT and hand OA in males (29). An association between visceral adiposity and the intensity of knee pain in patients diagnosed with knee OA was found to be positive by Li et al. (30). Zhang et al. revealed at the genetic level using Mendelian randomization that VAT is related to the prevalence of OA (31). In contrast to previous research, the strengths of the present study are reflected in the use of a nationally representative sample as well as a multicenter design, which provides a solid foundation for the generalizability and representativeness of the findings. In addition, this study utilized a large sample size, long-term follow-up, exhaustive data collection, advanced measurement techniques, and adjustment for multiple confounders, all of which contributed to the study’s precision and reliability. Although other studies have their unique strengths in specific aspects, such as the use of Mendelian randomization, the analysis of OA at multiple sites, and the conduct of sensitivity analyses, the present study is particularly notable for the breadth and representativeness of its sample.

For the final study, 3,779 participants from the NHANES 1999–2018 cohort were included in the analysis, of whom 516 had OA. Participants with OA had higher levels of VAI in comparison to normal individuals. This relationship still existed after adjusting for all covariates. In the RCS, we found a nonlinear positive correlation between VAI and OA prevalence. Among non-diabetics, females, races other than non-Hispanic, and those with less than high school, stratification effects were observed. Interaction tests showed that age, sex, BMI, education attainment, race, hypertension, and diabetes had no significant effect on this association.

OA is a degenerative disease mediated by inflammation, with inflammatory adipose tissue acting as a critical component (32, 33). Several studies have discovered that obese individuals have a higher likelihood of developing OA than normal individuals. VAT is a rich reservoir of pro-inflammatory cytokines and secretes a variety of adipokines, including IL-6 and leptin (34–36). IL-6 released from adipose tissue acts as a negative feedback regulator of osteoblast and osteoclast activation (37). Elevated levels of IL-6 have been reported to increase inflammatory responses and promote cartilage degradation (38). Increased concentrations of IL-6 in patients with OA confirm that they may exert an important contribution to the pathogenesis of OA (39, 40). Leptin secreted by adipose tissue regulates energy balance and body weight via a hypothalamic negative feedback loop (41). Studies have found a link between serum leptin levels and the development of OA. In an animal experiment in which rats were injected intra-articularly with leptin, Ait Eldjoudi et al. found that leptin modulated chondrocyte anabolism and correlated with the degree of cartilage destruction (42). In a diet-induced obese mouse study, increased serum leptin concentrations promoted the production of pro-inflammatory mediators and indirectly altered chondrocyte activity (43). Lipocalcin is a protective factor against OA and it regulates the metabolism of bone tissue (44). However, accumulation of VAT may inhibit lipocalin expression, thereby increasing systemic oxidative stress (45). The presented evidence implies that the correlation between visceral adiposity and an elevated prevalence of OA may be mediated by various mechanisms. Our findings further confirm that VAI is correlated with the likelihood of developing OA.

The main strengths of this study are the following: first, the study relies on a nationally representative sample of NHANES, which significantly enhances the generalizability of the findings. NHANES, as an annual surveillance system, has a national coverage of 5,000 people and includes data on a wide range of demographics, dietary habits, physical examinations, laboratory tests, and questionnaires. Second, the study employed statistical techniques such as weighted multivariate logistic regression analysis, RCS, subgroup analyses, and interaction tests, which helped to control for potential confounders and to probe deeply into the complex relationships among variables. Finally, the physical and serological indicators in the NHANES database were measured using standardized methods to ensure the robustness of the analysis.

However, there are several limitations to this study. First, causality could not be established due to the cross-sectional observational study design, which implies that potential effects that confounders may have on the study results could not be completely ruled out. Second, the diagnosis of arthritis relied heavily on self-report questionnaires, which may have led to diagnostic bias. In addition, the study population was predominantly European, so the findings may not be applicable to other ethnic groups. Further, despite adjusting for multiple covariates, there may have been uncontrolled confounders in the study, which may have affected the interpretation of the relationship between VAI and OA. Finally, due to the incomplete nature of the study data, this study was unable to conduct an analytic exercise consistent with *post hoc* sample size calculations to assess the power of the analytic sample.

The study showed that a higher visceral VAI was associated with a higher prevalence of osteoarthritis (OA) in older adults, which provides a new perspective on the relationship between obesity and osteoarthritis. Specifically, the study found a nonlinear positive correlation between elevated VAI and increased prevalence of OA, suggesting that the accumulation of visceral fat may play a key role in the pathogenesis of OA. This finding suggests that healthcare professionals should take visceral fat accumulation into account when assessing the prevalence of OA in older adults. In addition, the findings support the possibility that lowering visceral adiposity may reduce the prevalence of OA, which could inform the development of preventive and therapeutic strategies. For example, reducing visceral fat through dietary management and exercise may help reduce the prevalence of OA in older adults. The findings further support previous theories about the relationship between obesity and OA, namely that obesity, especially visceral obesity, is associated with an increased prevalence of OA. Previous studies have shown a positive correlation between obesity and OA, but the present study further refined this relationship by using VAI, a new gender-specific indicator. The findings provide new evidence for this theory, especially in the elderly population. Future studies should consider adopting more precise diagnostic methods and validating these findings in different populations.

## 5 Conclusion

In conclusion, our results indicate a potential correlation between a higher VAI and an elevated prevalence of OA in the elderly. Our findings may contribute to the understanding of the underlying mechanisms governing both VAI and OA. However, additional research and investigation are necessary due to the cross-sectional design of the NHANES.

## Data Availability

The datasets presented in this study can be found in online repositories. The names of the repository/repositories and accession number(s) can be found in the article/supplementary material.

## Glossary

OA: Osteoarthritis
NHANES.: National Health and Nutrition Examination Survey
VAT: Visceral adipose tissue
VAI: Visceral adiposity index
BMI: Body mass index
WC: Waist circumference
TG: Triglycerides
TC: Total Cholesterol
HDL-C: High-density lipoprotein cholesterol
RCS: Restricted cubic spline
Odds ratio: OR
Confidence interval: CI
LDL-C: Low-density lipoprotein cholesterol
TNF-alpha: Tumor necrosis factor-alpha
IL-6: Interleukin 6
METS-VF: Metabolic score of visceral fat
BRI: Body roundness index
*Ca*: Blood calcium
P: Blood phosphorus
BUN: Blood urea nitrogen
UA: Uric acid
Cr: Blood creatinine
